# Effect of Platelet Concentrates on Marginal Bone Loss of Immediate Implant Procedures: A Systematic Review and Meta-Analysis

**DOI:** 10.3390/ma14164582

**Published:** 2021-08-15

**Authors:** José González-Serrano, Carmen Vallina, Carlos González-Serrano, Andrés Sánchez-Monescillo, Jesús Torres, Gonzalo Hernández, Rosa María López-Pintor

**Affiliations:** 1 ORALMED Research Group, Department of Dental Clinical Specialties, School of Dentistry, Complutense University of Madrid, Plaza Ramón y Cajal s/n, 28040 Madrid, Spain; carmenvallinafkelly@hotmail.com (C.V.); jesust01@ucm.es (J.T.); ghervall@odon.ucm.es (G.H.); rmlopezp@ucm.es (R.M.L.-P.); 2 IDIBO Research Group, Stomatology Department, School of Health Sciences, Rey Juan Carlos University, Av. de Atenas, S/N, Alcorcón, 28922 Madrid, Spain; gonzalezserrano.carlos@gmail.com; 3 Division of Restorative Sciences, Norris Dental Science Center, Herman Ostrow School of Dentistry, University of Southern California, 925 W 34th Street, DEN 311, Los Angeles, CA 90089-0641, USA; asanchezmonescillo@gmail.com

**Keywords:** platelet concentrates, platelet-rich plasma, platelet-rich fibrin, immediate implants, marginal bone loss

## Abstract

Background: To evaluate marginal bone loss (MBL) in immediate implant procedures (IIP) placed in conjunction with platelet concentrates (PCs) compared to IIP without PCs. Methods: A search was performed in four databases. Clinical trials evaluating MBL of IIP placed with and without PCs were included. The random effects model was conducted for meta-analysis. Results: Eight clinical trials that evaluated MBL in millimeters were included. A total of 148 patients and 232 immediate implants were evaluated. The meta-analysis showed a statistically significant reduction on MBL of IIP placed with PCs when compared to the non-PCs group at 6 months (*p* < 0.00001) and 12 months (*p* < 0.00001) follow-ups. No statistically significant differences were observed on MBL of IIP when compared PCs + bone graft group vs. only bone grafting at 6 months (*p* = 0.51), and a significant higher MBL of IIP placed with PCs + bone graft when compared to only bone grafting at 12 months was found (*p* = 0.03). Conclusions: MBL of IIP at 6 and 12 months follow-ups is lower when PCs are applied in comparison to not placing PCs, which may lead to more predictable implant treatments in the medium term. However, MBL seems not to diminish when PCs + bone graft are applied when compared to only bone grafting.

## 1. Introduction

Nowadays, the placement of dental implants immediately after tooth extraction has become a hot topic [1]. Immediate implant procedures (IIP) not only decrease the total treatment time but also improve aesthetics and maintain soft tissues shape [2,3]. However, the dimension of the alveolar bone is also reduced even with IIP [4]. This bone resorption depends on several factors such as the thickness of the buccal wall and the gap size between bone and implant [5]. For these reasons, different substances such as bone grafts or platelet concentrates (PCs) have been proposed as alternatives to prevent this bone resorption [6,7].

PCs are autogenous substances derived from blood that consist essentially of supraphysiologic concentrations of platelets and growth factors [8]. Two types can be distinguished among PCs: platelet-rich fibrin (PRF) and platelet-rich plasma (PRP), which in turn can be rich in leucocytes (L-PRF and L-PRP) or pure (P-PRF and P-PRP) [9]. PCs can be prepared with or without red blood cells, and can be prepared from anticoagulated blood (PRP, P-PRF) or non-anticoagulated blood (L-PRF). Its preparation is carried out with centrifugation techniques that will depend on the size of the rotor, angulation and design of tubes, revolutions per minute or centrifugation time [10].

The high concentrations of growth factors and cytokines present in PCs are of great importance for tissue healing [8]. The efficacy of PCs in promoting wound healing and tissue regeneration is at the center of a recent academic debate [11]. PCs have demonstrated the capacity to improve soft tissue healing after surgical procedures [12,13,14]. There is also literature that confirms the local hemostatic efficacy of PCs after dental extractions in patients treated with antiplatelet drugs [15]. Nonetheless, there is still controversy about whether PCs have positive outcomes on hard tissue healing [16]. Indeed, several systematic reviews have shown moderate evidence supporting the clinical benefit of PRF on ridge preservation [17], or a favorable effect of L-PRF on bone regeneration and osseointegration [18]. Another study has shown insufficient evidence to establish the effectiveness of PCs in the prevention and treatment of medication-related osteonecrosis of the jaw [19].

Maintenance of peri-implant tissues play a main role in the functional sustainability of implant rehabilitation. Specifically, marginal bone loss (MBL) could compromise the long-term prognosis of implant procedures increasing the risk of peri-implantitis [20]. Although numerous surgical techniques have been developed to increase implant stability values [21,22], a recent study showed as primary implant stability is of main importance in the changes of marginal bone level during the early healing period [23]. Regarding peri-implant marginal bone, some authors have observed alveolar bone preservation related to IIP when PCs were applied [24,25], while Taschieri et al. [26] in a retrospective study did not find statistically significant differences in MBL of IIP placed with or without P-PRP with a follow-up of up to 5 years.

As there is controversy regarding the effect of PCs in hard tissue healing, and specifically in MBL after IIP [27], and no systematic review evaluating their effect have been performed up to now, we have conducted the first systematic review concerning the effectiveness of PCs on IIP for the maintenance of marginal bone. Therefore, the aim of this systematic review was to evaluate the evidence on MBL of IIP in combination with PCs when compared to IIP without using PCs.

## 2. Materials and Methods

This systematic review was structured according to the Preferred Reporting Items for Systematic Reviews and Meta-Analyses Protocols (PRISMA) statement [28], and it was recorded in PROSPERO (Registration number: CRD42021247128).

### 2.1. Focused Question

The aim of the study was to answer the following PICO (population, intervention, comparison, and outcome) question based on the PRISMA guidelines: In patients with at least one implant immediately placed after tooth extraction (population), what is the effectiveness of using PCs around the implant (intervention) when compared to not placing PCs (comparison) to diminish MBL (outcome)?

### 2.2. Eligibility Criteria

#### 2.2.1. Inclusion Criteria

The studies had to be (a) randomized controlled clinical trials (RCTs) or controlled clinical trials (CCTs), (b) published in English, and (c) performed only in humans. The population (P) had to be patients who had received dental implants immediately after tooth extraction placed with PCs (intervention group (I)) and without PCs (control group (C)) in a split mouth or a non-split mouth design. We also included studies in which bone substitutes were used, both in the group where PCs were applied, as well as in the control group. The following types of PCs were included: PRF, PRP, L-PRF, L-PRP, P-PRF, P-PRP and PRGF. The implants could have been placed in any location of the mandible and/or the maxilla and could have any length or width. With respect to the outcomes (O), the studies had to (a) radiographically assess the marginal bone resorption of the implants: MBL or crestal bone height, and (b) report the results in millimeters (mm) without restriction for follow-up time, in both the PCs and non-PCs groups.

#### 2.2.2. Exclusion Criteria

Studies excluded were: (a) studies that did not use PCs for IIP, (b) studies that evaluated PCs for conventional dental implant procedures (not placed immediately after tooth extraction), (c) studies that performed flapless procedures, and (d) non-randomized studies, review articles, experimental studies, retrospective studies, case reports, commentaries or letters to the Editor and unpublished articles.

### 2.3. Information Sources and Search Strategy

A comprehensive search of the literature was conducted without date restriction until 19 April 2021 in the following databases: PubMed/MEDLINE, The Cochrane Library, Web of Science and Scopus. The search was performed by two independent researchers (J.G-S., C.V.). The search strategy used was a combination of following keywords adapted to each database (Table 1).

### 2.4. Study Records

Two researchers (J.G-S. and C.V.) independently compared search results to ensure completeness and removed duplicates. Then, full title and abstract of the remaining papers were screened individually. Finally, full text articles to be included in this systematic review were selected according to the criteria described above. Disagreements over which eligible studies were to be included were discussed with a third reviewer (R.M.L-P.), and a consensus was reached. The reference lists of the included studies were also reviewed for possible inclusion. Agreement between reviewers was measured with the Kappa coefficient. The results were also expressed as the concordance between both reviewers (%).

### 2.5. Data Collection

Two independent reviewers (J.G-S. and C.V.) extracted the data. Data extraction included the following information: (a) the general characteristics of the selected studies: first author, year and country in which the study was conducted, type of study, number of patients and implants analyzed, sample age and sex, inclusion and exclusion criteria, the periodontal treatment received and the clinician who placed the implants (Table 2); (b) the surgical and implants characteristics of the included studies: types of PCs used, PCs preparation protocol, PCs application form, premedication, local anesthetic, surgical procedure, implant position, socket and gap characteristics, implant dimensions, regions of implants insertion, postsurgical medication, prosthetic procedure, marginal bone evaluation and complications (Table 3); and (c) MBL (mean ± standard deviation (SD)), implant survival rates (%) and follow-up period (months) of IIP placed with PCs and without PCs, respectively (Table 4).

### 2.6. Risk of Bias in Individual Studies

Two independent reviewers (J.G-S. and C.V.) evaluated the methodological quality of eligible studies following the Cochrane Collaboration’s tool for assessing risk of bias, which incorporates 7 domains [29]. The studies were classified as low risk of bias (low risk of bias for all key domains), unclear risk of bias (unclear risk of bias for one or more key domains), and high risk of bias (high risk of bias for one or more key domains) [29].

### 2.7. Assessment of Evidence Levels

We evaluated the quality of evidence using the Grading of Recommendations Assessment, Development and Evaluation (GRADE) framework, which characterizes the quality of a body of evidence based on the study limitations, imprecision, heterogeneity and inconsistency, indirectness, and publication bias. The GRADE approach enabled us to assign one of four confidence levels (high, moderate, low, or very low) [30].

### 2.8. Synthesis of Results

Review Manager (RevMan) (Computer program), version 5.3 (The Nordic Cochrane Centre, The Cochrane Collaboration, Copenhagen, Denmark, 2014) was used to perform the meta-analysis. The Cochrane Q-test was used to assess heterogeneity between studies. I^2^ index was calculated to perform quantitative analysis of heterogeneity, which assesses the percentage of variation in the global estimate attributable to heterogeneity (I^2^ = 25%: low; I^2^ = 50%: moderate; I^2^ = 75%: high heterogeneity). The random effect model was used to group the study-specific estimates. Publication bias was planned to be conducted in analysis with 10 studies or more [31]. Statistical significance was defined as a *p* value < 0.05.

### 2.9. Sensitivity Analysis

For sensitivity analysis, each meta-analysis was performed using all possible combinations, including fixed-effect methods and standardized mean difference (SMD).

## 3. Results

### 3.1. Study Selection

**Figure 1:**
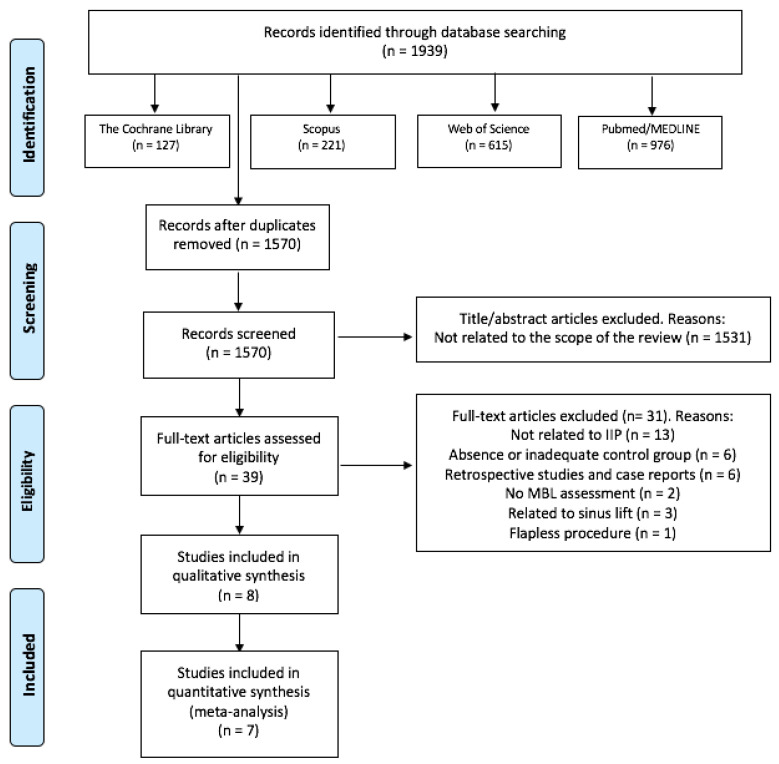
Flow diagram of the literature search, according to the Preferred Reporting Items for Systematic Reviews and Meta-Analyses (PRISMA).

The search strategy resulted in 1939 results, of which 1570 remained after removing the duplicates. Then, two independent researchers (J.G-S. and C.V.) reviewed all the titles and abstracts and excluded 1531 papers that were outside of the scope of this review. Thus, we obtained 39 potential references. After reading the full text of those 39 papers, 13 were discarded for not analyzing IIP, 6 for an absence or inadequate control group, 6 for being retrospective observational studies or case reports, 3 studies related to sinus lift, two for not assessing MBL and one for performing flapless procedures. Finally, 8 studies were included in our systematic review [32,33,34,35,36,37,38,39] (Figure 1). Only two of the three groups of Alam et al. [38] study were considered in this review (PCs + bone graft group vs. only bone graft group). There was a 99.18% concordance between the two authors with a Kappa coefficient of 0.85 (SE 0.04, 95%CI [0.77, 0.93]) for titles and abstracts, and a 97.43% concordance with a Kappa coefficient of 0.93 (SE 0.08, 95%CI [0.74, 1]) for full-text articles, respectively.

### 3.2. Study Characteristics

The selected articles were published between 2015 and 2020 [32,33,34,35,36,37,38,39]. One study was a CCT [32] and seven studies were RCTS [33,34,35,36,37,38,39]. Three of the selected studies had a split-mouth design [32,33,37]. Four of them were performed in India [35,36,38,39], one in Syria [32], one in Saudi Arabia [33], one in Turkey [37] and one in Egypt [39] (Table 2).

#### 3.2.1. Patients’ Characteristics

Combining the samples from each study, a total of 148 patients were studied. Only three studies reported the age and sex of the included patients [32,36,39]. The age ranged from 18 to 55 years, there being 32 males and 23 females. Systemically healthy patients were included in every study [32,33,34,35,36,37,38,39]. Regarding the inclusion of smokers, six studies excluded smokers [32,33,35,37,38,39], one included smokers of less than 10 cigarettes per day [36] and one included smokers [34]. Concerning the periodontal status, Al Nashar et al. [32] included patients with chronic periodontitis in the mandibular anterior region, Khan et al. [35] excluded patients with severe periodontal bone loss, and six studies [33,34,36,37,38,39] included patients with good oral hygiene. The patients of four of the selected studies underwent periodontal therapy prior to IIP [33,35,37,39], one study included patients for professional periodontal maintenance during the study [37], another study evaluated oral hygiene during the study [38], while the other two studies did not mention it [32,34] (Table 2).

#### 3.2.2. Implants and Bone Characteristics

All the included studies used IIP in both the study and the control groups [32,33,34,35,36,37,38,39]. A total of 232 IIP (117 implants with PCs and 115 implants without PCs) were studied. Four of the included studies used PCs (study group) vs. no PCs (control group) [32,34,35,36,37], and four of them used PCs in combination with bone substitutes (study group) vs. only bone substitutes (control group), of which 3 used xenografts [33,37,39] and one used a synthetic bone graft [38] for IIP. The implants were placed in the mandibular lateral incisors [32], in the maxillary anterior teeth [33,37,38] and premolars [39], and different sites of maxilla and mandible [34,35,36]. Two studies placed the implants at crestal bone level [32,33], one study slightly below the bone crest [35], two studies below the alveolar bone: 2 mm [36] and 1 mm [39], one study 2 mm below the cementoenamel junction of the adjacent teeth [38], while Gangwar et al. [34] did not report it. With respect to the sockets included in the selected studies, two studies reported four bone walls [32,35], two studies buccal bone loss [33,37] and four studies adequate bone [34,36,38,39]. Only three studies mentioned the bone–implant gap: Khan et al. [35] included gaps <2 mm, Öncü et al. [36] included gaps of about 1 mm and Alam et al. [38] included gaps between 2 and 5 mm (Table 3).

#### 3.2.3. Radiographic Evaluation

Al Nashar et al. [32] used orthopantomography to determine MBL, while the other seven studies [33,34,35,36,37,38,39] used periapical radiographs. Six of them [34,35,36,37,38,39] used positioners, so that the radiographs were reproducible in the different follow-ups, while ArRejaie et al. [33] did not specify it.

Seven of the included studies used the real length of the implants to calculate the radiographic MBL [32,34,35,36,37,38,39] from the shoulder of the implant [32,34,35,36,37,38] and the implant–abutment connection [39] to the first bone–implant contact site. ArRejaie et al. [33] measured MBL but did not specify how. All the studies expressed the results in millimeters [32,33,34,35,36,37,38,39]. Six of them expressed the values in the mesial and distal aspects [33,34,35,37,38,39], while two of them [32,36] expressed the mean values between mesial and distal aspects (Table 3).

#### 3.2.4. Platelet Concentrates (PCs) Protocols

Three of the included studies used PRP [32,33,34], while the other five used PRF [33,34,35,36,37,38,39]. Two studies applied PCs as liquid [32,34], one as gel form [33], one as both liquid and solid [35], and four studies as solid membranes [36,37,38,39]. The different protocols of the studies included are shown in Table 3.

### 3.3. Main Findings

The results of MBL, implant survival rates and follow-up periods of the included studies are shown in Table 4.

#### 3.3.1. Marginal Bone Loss (MBL)

- PCs vs. non-PCs

Al Nashar et al. [32] reported significantly lower MBL of IIP placed with PCs in comparison to control group at 3 months (*p* < 0.0001), 6 months (*p* < 0.0001) and 12 months (*p* < 0.0001) follow-ups. Gangwar et al. [34] reported statistically lower MBL at 6 months follow-up of PCs group in comparison to control group (mesial: *p* = 0.003, distal *p* = 0.001). Khan et al. [35] achieved no statistically significant differences in MBL between two groups at any of the visits during the 13–14 months follow-up period. Öncü et al. [36] found statistically lower MBL in the PCs group in comparison to the control group at 12 months follow-up (*p* ≤ 0.05).

- PCs + bone graft vs. only bone graft

ArRejaie et al. [33] showed no statistically significant differences between the two groups in MBL at 3 months follow-up (*p* = 0.067), but statistically significant reductions at 6 months (*p* < 0.01), 9 months (*p* < 0.0001), and 12 months (*p* < 0.0001) follow-ups in PCs in comparison to control group. Soni et al. [37] showed no statistically differences in MBL at 4 months in the PCs group when compared to control group (mesial: *p* = 0.85; distal: *p* = 0.94). Alam et al. [38] did not find statistically significant differences in MBL between groups at 12 months in either mesial or distal aspects (*p* > 0.05). Abdel-Rahman et al. [39] reported no statistical differences in MBL at 6 months (*p* = 0.11), 12 months (0.078) and 18 months (0.052) after IIP between the two groups.

#### 3.3.2. Implant Survival Rates

Six studies achieved 100% survival rates in each group [32,35,36,37,38,39]. ArRejaie et al. [33] and Gangwar et al. [34] did not specify the survival rates in each group. Gangwar et al. [34] reported a 90% survival rate for the two groups together.

### 3.4. Risk of Bias within Studies

According to the 7 domains for assessing risk of bias, we established that all the included studies had a high risk of bias (high risk of bias for one or more domains) (Table 5). One study [32] had a high risk of selection bias because it did not mention if randomization was made and because no allocation concealment was made. Four studies had an unclear risk of selection bias because they did not report how the allocation concealment was performed [34,36,37,38,39]. All the clinical trials included [32,33,34,35,36,37,38,39] had high risk of performance bias because the surgeon had to know what technique was performing. Five studies [33,34,36,37,39] had an unclear risk of detection bias because they did not specify who collected the data. Three studies had an unclear risk of attrition bias as no sex and age data were reported [33,34] and three patients in each group were lost to 12-months follow-up [38], respectively. Two of them [33,34] had unclear risk of reporting bias as no implant survival rates were reported in each group, and one study [37] had a high risk of reporting bias because it showed negative results for MBL.

### 3.5. Evaluation of Evidence Levels

According to the GRADE framework, the quality of the evidence related to the overall ranking of efficacy was low because of the low number of studies included in the meta-analysis, the high risk of bias in all the included studies or the lack of publication bias. There was low-quality evidence in the comparisons between PCs and non-PCs, and the comparisons among PCs + bone graft and bone grafting alone, respectively.

### 3.6. Synthesis of Results: Meta-Analysis

We used mean ± SD of the implants MBL in mm as the main effect measure. In those studies that showed the mean and standard error of mean (SEM), the SD was calculated by multiplying the standard error of a mean by the square root of the sample size [33,35]. Also, the formulae for combining groups was used in those studies that only showed the results of the mesial and distal aspects of the implants [33,34,35,37,38,39]. Finally, seven articles were included for meta-analysis [32,33,34,35,36,38,39]. Al Nashar et al. [32], Gangwar et al. [34] and Khan et al. [35] studies were included for the MBL after 6 months follow-up, and Al Nashar et al. [32], Khan et al. [35] and Öncü et al. [36] studies were included for the MBL after 12 months follow-up in PCs group vs. non-PCs group, respectively. ArRejaie et al. [33], Alam et al. [38] and Abdel-Rahman et al. [39] studies were included for the MBL after 6- and 12-month follow-ups in PCs + bone graft group vs. only bone graft group.

**Figure 2:**
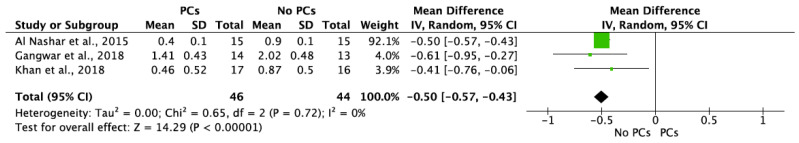
Random-effect meta-analysis evaluating marginal bone loss (MBL) in both aspects at 6 months in immediate implants procedures with PCs vs. no PCs.

**Figure 3:**
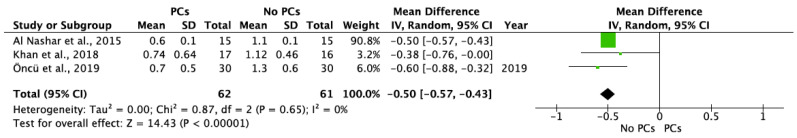
Random-effect meta-analysis evaluating MBL in both aspects at 12 months in immediate implants procedures with PCs vs. no PCs.

**Figure 4:**
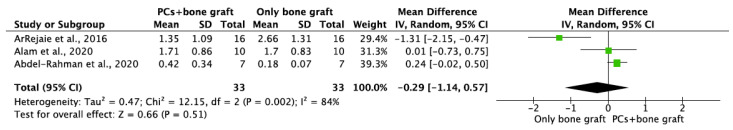
Random-effect meta-analysis evaluating MBL in both aspects at 6 months in immediate implants procedures with PCs + bone graft vs. only bone graft.

**Figure 5:**
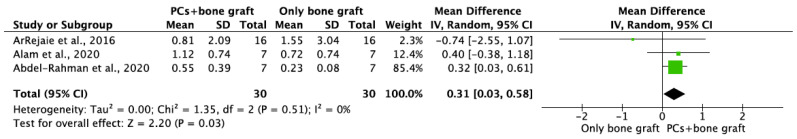
Random-effect meta-analysis evaluating MBL in both aspects at 12 months in immediate implants procedures with PCs + bone graft vs. only bone graft.

MBL in both aspects after 6 and 12 months of IIP with PCs vs. non-PCs and PCs + bone graft vs. only bone graft were evaluated by mean differences and 95% confidence intervals, respectively. The results of the meta-analysis showed that MBL at 6 months follow-up was significantly lower in IIP placed with PCs in comparison to procedures without PCs (MD −0.50, 95%CI [−0.57, −0.43]; *p* < 0.00001) (Figure 2). The random-effect model suggested a low heterogeneity across the controlled trials (I^2^ = 0%) (Figure 2). It also showed that MBL at 12 months follow-up was significantly lower in IIP placed with PCs when compared to non-PCs group (MD −0.50, 95%CI [−0.57, −0.43]; *p* < 0.00001) (Figure 3). The random-effect model also suggested a low heterogeneity across the controlled trials (I^2^ = 0%) (Figure 3). At 6 months, the meta-analysis showed that MBL was not statistically significant when compared to the PCs + bone graft group vs. only bone graft (MD −0.29, 95%CI [−1.14, −0.57]; *p* = 0.51) (Figure 4). The random-effect model showed a high heterogeneity across the controlled trials (I^2^ = 84%) (Figure 4). However, MBL at 12 months was statistically higher in the PCs + bone graft group when compared to only the bone graft group (MD 0.31, 95%CI [0.03, 0.58]; *p* = 0.03) (Figure 5). The random-effect model showed a low heterogeneity across the controlled trials (I^2^ = 0%) (Figure 5).

### 3.7. Sensitivity Analysis

The results of the different meta-analyses are contained in the Supplementary Material (Figures S1–S12). MBL at 12 months in PCs + bone graft vs. only bone graft showed statistically significant differences when MD was used, and no statistically significant differences when SMD was applied. In the other meta-analyses, the statically significant results did not change and only I^2^ values were different depending on which method was used.

## 4. Discussion

### 4.1. Summary of Evidence

This systematic review included eight clinical trials [32,33,34,35,36,37,38,39] evaluating MBL of IIP placed with and without PCs, and 7 of them [32,33,34,35,36,38,39] were evaluated by meta-analysis. Although there are not many clinical trials on this subject and, therefore, these results should be taken with caution, the meta-analysis determined that MBL of IIP is statistically lower when PCs are applied in comparison to not placing PCs at 6-month and 12-month follow-ups. However, MBL was not statistically significant in the PCs + bone graft group when compared to only bone grafting at 6 months, and statistically higher MBL was found at 12 months in the PCs + bone graft group when compared to only bone grafting. Nonetheless, the results of this last meta-analysis should be interpreted with caution due to the results obtained in the sensitivity analysis and, therefore, more studies are needed to try to clarify this fact.

PCs seem to prevent MBL in IIP, which may lead to more predictable implant treatments in the medium term. This may be explained by the fact that PCs present antimicrobial properties and may help soft tissues to heal faster [40], thus preventing the initial MBL. Another reason that explains this result is that bone–implant contact has been histologically seen to be increased twofold in implants when PRF is used [41], or to have an 84.7% of bone–implant contact with the application of PRGF [42]. Moreover, a clinical study showed faster osseointegration in non-immediate implants placed with PRF in comparison to control group [43]. However, Taschieri et al. [26] in a retrospective study found no statistically significant differences between the use of P-PRP vs. non-use in IIP with a follow-up of up to 5 years. This fact may indicate that the application of PCs in IIP may not have a long-term benefit on MBL. On the other hand, when PCs are used in combination with a bone graft, this seems to have no benefit on MBL when compared to bone grafting alone. Nonetheless, this should be taken with caution because this meta-analysis showed high heterogeneity between the studies [33,38,39] and one of them used synthetic bone graft [38], while the others used xenografts [33,39]. Further studies focusing on this fact are needed to evaluate whether PCs in combination with bone graft have benefits in reducing MBL when compared to only bone grafting.

Regarding survival rates of IIP, this systematic review showed the same survival results when PCs were applied (100%) in comparison to the non-PCs group (100%) with at least 12 months follow-up [32,35,36,38,39]. The survival rates observed in this systematic review in both groups are high and comparable to a retrospective study that evaluated 1139 immediately loaded implants placed with PRGF, that achieved more than 96.8% survival rates with a mean follow-up of 28 months [44]. Nonetheless, further randomized controlled trials with larger sample sizes are needed to corroborate these findings.

### 4.2. Strength and Limitations

This systematic review has some limitations. Only RCTs and CCTs were included. Although these studies have the highest methodological quality, some information may have been lost from other studies conducted with a different design. Although most of the best journals only publish studies in English, not including manuscripts in other languages may imply language bias. The included trials presented limited samples and high risk of bias. Also, all the meta-analyses included a maximum of 3 articles, and in three of them there was one study that clearly presented the highest weight [32,39]. Another limitation is the different nomenclatures and protocols to obtain PCs that are available in the literature. In the present systematic review, both the studies that used PRP [32,33,34], as well as those studies using PRF [35,36,37,38,39], used different preparation procedures with respect to revolutions per minute and centrifugation time. Moreover, future studies should specify the size of the rotor, the angulation and design of tubes [10]. However, a previous systematic review concluded that there are not significant differences regardless of the PCs to be used [45].

Regarding the radiograph technique used to measure MBL, Al Nashar et al. [32] used orthopantomography, which is not the best way to assess interproximal bone levels. However, we decided to include this study because they considered the magnification factor in the measurements, and it has also been used to analyze the fractal dimension of the trabecular peri-implant bone [46]. The rest of studies used periapical radiographs, which are more suitable to measure MBL. It should be noted that these radiographs should be as reproducible as possible so as not to bias the results, which requires the use of positioners. Nonetheless, these radiographic techniques are limited to mesial and distal aspects, and it is, therefore, not possible to assess the buccal bone, where the resorption is always greater after a tooth extraction [47,48]. Future studies may also use three-dimensional analysis to measure this bone loss around implants [33,49]. Despite these limitations, the meta-analysis showed statistically lower MBL when PCs are applied compared to non-placing PCs for IIP with a low heterogeneity between the studies.

## 5. Conclusions

In conclusion, with a low certainty of evidence, PCs show a moderate effect in reducing MBL of IIPs when compared to not placing PCs. With a low certainty of evidence, PCs + bone graft show a small unimportant effect or no effect on MBL of IIPs when compared to bone grafting alone. Further randomized clinical studies are needed to confirm these results.

## Figures and Tables

**Table 1:** Search strategy employed in the present systematic review.

**Keywords Employed Using Boolean Operator “OR”**	**Boolean** **Operator “AND”**	**Keywords Employed Using** **Boolean Operator “OR”**
“Immediate implant”, “immediate implantation”, “post-extraction implant”, “immediate implant procedure”, “immediate implant placement”		“Platelet concentrates”, “platelet-rich fibrin”, “PRF”, “platelet-rich plasma”, “PRP”, “leukocyte platelet-rich fibrin”, “L-PRF”, “leukocyte platelet-rich plasma”, “L-PRP”, “pure platelet-rich fibrin”, “P-PRF”, “pure platelet-rich plasma”, “P-PRP”, “plasma-rich in growth factors”, “PRGF”, “injectable platelet-rich fibrin”, “I-PRF”, “growth factors”, “platelet-derived growth factors”

**Table 2:** General characteristics of the selected studies.

Author, Year	Country	Type of Study	Patients	Implants		Age (Years)	Sex	Inclusion Criteria	Exclusion Criteria	Periodontal Therapy	Clinician
				PCs	Non-PCs						
**Al Nashar** **et al., 2015**	Syria	CCT with split-mouth design	15	15	15	30–55	Male: 7 Female: 8	Patients with good health, with no chronic disease, non-smoking, physically able, with chronic periodontitis in the anterior region of the mandible which had lost 75% of the supporting bone or had a probing depth >8 mm of four bony walls of the remaining alveolus with at least 5 mm depth on both sides and the presence of 5 mm of bone beyond root apex	Patients with any disease, condition, or medication that might compromise healing or osseointegration, unable/unwilling to return for follow-up visits, or needing grafting of the implant site	NA	NA
**ArRejaie et al., 2016**	Saudi Arabia	RCT with split-mouth design	16	16	16	NA	NA	Patients with either tooth fracture, endodontic failure, or badly decayed teeth in the anterior and premolar regions of the maxilla with previous buccal bone loss	Patients with systemic contraindications to treatment, pregnants, smokers, having received systemic antibiotics or non-steroidal anti-inflammatory drugs within the last 3 months prior to treatment, or having received surgical treatment in the selected sites within the year before the initiation of the study	Oral hygiene instructions and SRP Surgical phase: < 10% O’Leary Plaque Index	The same surgeon
**Gangwar et al., 2018**	India	RCT	27	14	13	NA	NA	Patients >18 years with missing maxillary or mandibular tooth, adequate bone volume to accommodate an implant of appropriate size and with good oral hygiene	Pathological radiolucency in jaw bone of more than 1 cm, chronic inflammatory rheumatoid disease, uncontrolled diabetes, osteoporosis, Systemic corticosteroid treatment of more than 1 month within 1 year, and severe disease with a life expectancy <1 year	NA	NA
**Khan et al., 2018**	India	RCT	14	17	16	PCs 32.59 ± 1.65 Non-PCs 33.25 ± 1.83	NA	Patients > 18 years, ASA I, non-smokers (>2 months), with monoradicular tooth indicated for extraction (trauma, endodontic failure and non-restorable carious lesion, root/crown fracture), intact bone in all dimensions, jumping gap < 2 mm, with a suitable occlusion	Pregnant women or nursing mother, smokers, patients with localised infection (presence of chronic pain, purulent periodontal and endodontic lesion, severe periodontal bone loss with a remaining alveolar height < 7 mm), Patients taking medication that may affect the clinical outcome in last 6 months, extraction site of mobile teeth	Preliminary phase: hygiene instructions, SRP Before surgical phase: complete SRP and polishing	NA
**Öncü et al., 2019**	Turkey	RCT with split-mouth design	26	30	30	40.2 ± 11.5	Male 16 Female 10	No systemic health problems; no need of sinus floor augmentation, distraction osteogenesis, or bone grafting, and having at least two adjacent or contralateral premolar or molar teeth that needed extraction in the mandible/maxilla	Insufficient bone volume, parafunctional habits, smoking more than 10 cigarettes per day, systemic disorders, and poor oral hygiene	During the study, patients enrolled in an individually maintenance care program for professional cleaning and examinations	The same surgeon
**Soni et al., 2020**	India	RCT	16	8	8	PCs: 21–45 Non-PCs: 18–45	NA	Patients >18 years; good oral hygiene and satisfactory periodontal status of the remaining dentition; presence of a single failing tooth in anterior maxilla; patients who gave positive informed consent and patients available for follow-up	Metabolic or systemic disease affecting the integration of implant or connective tissue health surrounding implant; history of irradiation in the head-and-neck area; smokers; pregnant women; parafunctional habits such as bruxism, tongue thrust, and teeth clenching; untreated generalised periodontitis; psychiatric disorders or unrealistic expectations and acute infection (abscess) at the intended site for implant placement	Initial periodontal therapy was done before the surgical procedure	NA
**Alam et al., 2020**	India	RCT	20 *	10	10	18–45	NA	Patients with single maxillary anterior tooth with poor prognosis and indicated for extraction (root fractures, endodontic failures, root caries, internal or external resorption, and over retained deciduous tooth); with good oral hygiene and periodontal status of remaining dentition; without any metabolic and systemic disease; with sufficient quality and quantity of bone and available for follow-up visits.	Teeth with associated periapical pathology, patients with parafunctional habits, history of smoking, diabetes, any other systemic health problems, immunocompromised state, pregnant females, peri-implant gaps <2 mm or >5 mm and primary stability <35 N/cm.	Patients recalled at regular intervals to evaluate oral hygiene	The same surgeon
**Abdel-Rahman et al., 2020**	Egypt	CCT	14	7	7	22.54 ± 4.97 (18–36)	Male: 9 Female: 5	Patients needing a extraction of a non-restorable maxillary incisor or premolar, with adequate horizontal and vertical bone, with opposing occlusion, good oral hygiene and no medical limiting conditions.	Teeth adjacent to the future implant that are periodontally or endodontically compromised, no opposing dentition; inadequate oral hygiene; chronic medical conditions (hemorrhagic disease, uncontrolled diabetes), smokers, alcohol abusers and bruxers.	All patients were instructed for oral hygiene and received adequate periodontal scaling prior to implant placement	NA

RCT: randomized controlled trial; CCT: controlled clinical trial; NA: not available; PCs: platelet concentrates; SRP: scaling and root planning; * only 2 of 3 groups of this study were selected in this review.

**Table 3:** Surgical and implants characteristics of the included studies.

Author, Year	Al Nashar et al., 2015	ArRejaie et al., 2016	Gangwar et al., 2018	Khan et al., 2018	Öncü et al., 2019	Soni et al., 2020	Alam et al., 2020	Abdel-Rahman et al., 2020
**Types of PCs**	PRGF	PRP	PRGF	PRF	L-PRF	A-PRF	L-PRF	PRF
**PCs preparation protocol**	10 mL of peripheral blood 3.8% trisodium citrate 270 rpm for 7 min (PRGF System; BTI, Álava, Spain) The middle layer was collected Leukocytes were not collected 50 µL of 10% CaCl_2_ solution was added to coagulation	10 mL of blood 0.5 mL sodium citrate 200 G for 20 min Plasma: 400 G for 10 min Bovine thrombin and 10% calcium chloride were added to coagulation	8 mL of blood 0.2 mL of 3.2% sodium citrate 270 G (3500 rpm) for 10 min PRGF located above the red clot was used	10 mL of blood without any anticoagulant 3000 rpm for 12 min (REMI, Laboratories, India). PRF in central clot was collected	9 mL of blood without anti-clotting agent (Becton Dickinson Vacutainer) 2700 rpm for 12 min (PC-02, Process Ltd., Nice, France) The middle fibrin clot was transferred to the L-PRF box (Process Ltd., Nice, France) and compressed to obtain L-PRF membranes	10 mL tubes No anticoagulant Centrifugation (DUO Quattro PRF Centrifuge, Nice, France) at 1300 rpm for 8 min. The middle layer was collected and placed in the PRF box for A-PRF membrane formation	5 mL of venous blood Centrifuge machine (REMI R-8C, Maharashtra, India) PRF was obtained	10 mL tubes No anticoagulant Centrifuged at 3000 rpm and 400 g for 10 min. The middle layer was collected for PRF membranes formation
**PCs application form**	Liquid: PRGF injected into the drill holes immediately before implant placement. Implants were dipped in PRGF before seating	Gel: PRP gel combined with bovine-derived xenograft	Liquid: Implants dipped in PRGF before placing it	Liquid and solid: Implants bio-activated and covered with PRF membrane	Solid: L-PRF membrane applied inside the implant cavity	Solid: Xenograft was placed into the defects and then covered with A-PRF membranes	Solid: L-PRF combined with synthetic bone graft	Solid: PRF membranes combined with bovine-derived xenograft
**Premedication**	600 mg clindamycin 1 h before surgery	NA	NA	500 mg amoxicillin and 125 mg of clavulanic acid a day before surgery	NA	2 g amoxicillin with potassium clavulanate (augmentin) 1 h before surgery, and rinse with chlorhexidine (0.2%) for 1 min before intervention.	2 g amoxicillin with potassium clavulanate	NA
**Local anesthetic**	3.6–5.4 mL of mepivacaine HCl 2% with vasoconstrictor (levonordefrin) 1:20.000	NA	2% xylocaine hydrochloride with 1:20.0000 adrenaline	2% xylocaine, with 1:80.000 adrenaline	Ultracaine	2% lignocaine, with 1:80.000 adrenaline	Lignocaine 2% with 1:200.000 adrenaline	Local anesthesia
**Surgical procedure**	Full-thickness flap Teeth extracted gently with minimum trauma Sockets carefully debrided and irrigated with sterile saline. Osteotomies according to standard protocols, with slow-speed sequential drills and copious irrigation Closure of the wound was obtained by coronal repositioning of the flap	Full-thickness flap Vertical releasing incisions when needed. Teeth carefully extracted Infected granulation tissues were removed All implants were placed completely into the extraction socket with primary stability	Mucoperiosteal flap reflected. Extraction was performed with the help of root forceps, bur, periotome, etc. Drilling procedure 2 mm beyond the apex of tooth, with internal irrigation. Flap sutured to achieve primary tension-free closure	Full-thickness flap. Atraumatic extractions. Socket debrided using surgical curette. Implants placed at a speed of 30 rpm. All implants had a primary stability of at least 35 N/cm	Mucoperiosteal flap. Carefully extractions. Sockets cleaned and rinsed with saline. The implant sites 5 mm apart. Flap sutures to original position. Healing caps were not covered	Crestal incision with releasing incision Full-thickness mucoperiosteal flap Tooth extracted and socket debrided and irrigated with saline Implants placed with cover screws Primary closure of the wound	A traumatic extraction, socket cleaned and irrigated. Implants placed in the palatal wall and left for submerged healing. Primary closure	Intrasulcular incision full thickness mucoperiosteal flap Atraumatic extraction Socket curetted and irrigated with saline Implant placed with a customised surgical guide. Implants placed 2–3 mm past the extracted tooth apex Cover screw and primary closure with 4.0 silk suture
**Implant position**	At the crestal ridge	At the crestal bone level	NA	Slightly below the bone crest level	Submerged 2 mm below the margins of the socket	NA	2 mm below the line joining the cementum-enamel junction of adjacent teeth	1 mm sub crestal in all cases
**Inclusion sockets**	Presence of four bony walls	Buccal bone loss	Adequate bone volume	All four walls intact	Sufficient bone volume	Buccal bone defect	Sufficient quantity of bone	Adequate horizontal and vertical bone
**Included gaps**	NA	NA	NA	>2 mm	About 1 mm	NA	Between 2–5 mm	Nr
**Implants characteristics**	Length: 10–12 mm Diameter: 3.6 mm (Euroteknika, Sallanches, France)	Length: 10–14 mm Diameter: 3.4–3.8 mm (Friadent, Dentsply)	NA	The Myriad Plus (MyriadTM Plus Implant System)	Length: 12 mm Diameter: 4.1 mm (ITI SLActive, Straumann)	Double piece, (ADIN; Touareg™-S)	Tapered implants with internal trilobed (Myriad plus, Equinox Medical Technologies B.V, Netherlands)	Length: 14 mm Diameter: 3.6, 4 and 4.5 mm (Dentium system, Superline, Seoul, Korea)
**Regions of implants insertion**	Mandibular lateral incisors	Maxillary anterior and premolar regions	Anterior and posterior maxilla. Anterior and posterior mandible	PCs: 5 maxilla, 12 mandible Non-PCs: 5 maxilla, 11 mandible	PCs: 12 maxilla; 18 mandible Non-PCs: 14 maxilla, 16 mandible	All in the anterior maxilla	Maxillary anterior region	Maxillary incisors and premolars
**Postsurgical medication**	0.2% chlorhexidine HCl twice daily for 7 days 300 mg clindamycin orally every 6 h for 5 days and ibuprofen 600 mg twice daily for 7–10 days	NA	The patient is discharged after prescribing antibiotic and analgesic and hexidine mouthwash. The patient was seen after 7 days postoperatively for suture removal	500 mg amoxicillin and 125 mg clavulanate acid twice daily during 6 days, Zerodol every 12 h and 0.2% chlorhexine mouth wash	1 g amoxicillin and clavulanic acid 2 times/day, flurbiprofen 2 times/day and chlorhexidine gluconate 3 times/day	Antibiotics, analgesics, anti-inflammatory and chlorhexidine mouth rinses for 7 days	400 mg ibuprofen and 325 mg paracetamol 3 times per day for 3 days	500 mg amoxicillin every 8 h for 1 week
**Prosthetic procedure**	Healing period of 3 months. Then, healing abutments were placed. Prosthetic rehabilitation started 2 weeks later, where crowns were cemented with temporary cement	After 6 months, healing abutments were connected to the implants, and the prosthetic procedures were performed. Crowns were cemented using implant cement materials	NA	NA	The healing caps were placed in the 3rd month	Second-stage: 4 months Healing cap placed for 2 weeks	Second-stage: 3 months Finally restored with porcelain fused to metal crowns, luted with temporary cement	Second-stage: 6 months Healing cap placed for 2 weeks 1 week later: final cemented crown
**MBL evaluation**	An independent radiologist blinded to the study groups Digital panoramic radiographs The implant length fixture was measured and compared to the real implant length to determine the magnification factor in the image	A standardised long cone parallel technique was used to record the radiographic parameters. Three CBCT images (baseline, 6 and 12 months post-surgery)	Intraoral periapical radiograph with XCP extension cone paralleling film-holding device. The implant-abutment junction was used as a reference point for all measurements	An independent investigator unaware of the treatment modality. Radiovisiography (RVG) The images were calibrated geometrically based on implant length. The radiograph was obtained in a constant and reproducible plane, using film holder and a template	Periapical radiographs using long cone paralleling technique and employing a positioner (X-ray Holders, KerrHawe). Upper corner of the coronal shoulder of the implant as reference point. Measurements from reference point to the first bone–implant contact. Image J software (version 1.49 m, National Institutes of Health)	Periapical radiographs using a digital intraoral sensor (Sirona Dental system, Bensheim, Germany), an X-ray positioner with an individually customised acrylic positioning jig Measurements with Sirona software from the shoulder of the implant to the first bone–implant contact Images were calibrated with the known size of implant	Periapical radiographs taken with long cone paralleling technique using positioner The images were calibrated by the known length of the dental implant Distance from the implant shoulder to the first bone-to implant was measured using contact Image J software (1.47 V Wayne Rasband, National Institutes of Health, Bethesda, MD, USA) by two calibrated examiners	Periapical radiographs taken with the long cone parallel technique, using Rinn XCP (Dentsply, Friadent Schweiz, Nidau, Switzerland) and a customised bite-block. The known implant length was used as reference. The distance from the implant–abutment connection to the marginal bone level was measured. Digital tracing was conducted with Scanora 5.2 software (Tuusula, Finland)
**Complications**	without infections or complications	NA	NA	NA	No complications were observed.	Only one patient with cover screw exposure	NA	No signs of dehiscence, infection or mobility

NA: not available.

**Table 4:** Marginal bone loss (MBL) during the postoperative period (mean ± SD) and implants survival rates (%).

Marginal Bone Loss (mm)										Survival Rates (%)	
			PCs			No PCs				PCs	No PCs
	Follow-Up After IIP (Months)	Implants (n)	Mesial Aspect	Distal Aspect	Both Aspects	Implants (n)	Mesial Aspect	Distal Aspect	Both Aspects		
**Al Nashar et al., 2015**	3 6 12	15	-		0.2 ± 0.1 0.4 ± 0.1 0.6 ± 0.1	15	-		0.6 ± 0.0 0.9 ± 0.1 1.1 ± 0.1	15/15 (100%)	15/15 (100%)
**ArRejaie et al., 2016**	3 6 9 12	16	1.66 ± 0.96 ^†^ 1.30 ± 0.96 ^†^ 0.83 ± 1.16 ^†^ 0.80 ± 0.96 ^†^	1.76 ± 0.96 ^†^ 1.40 ± 1.24 ^†^ 0.87 ± 1.16 ^†^ 0.82 ± 2.84 ^†^	1.71 ± 0.95 * 1.35 ± 1.09 * 0.85 ± 1.14 * 0.81 ± 2.09 *	16	2.27 ± 1.8 ^†^ 2.76 ± 1.24 ^†^ 1.66 ± 1.12 ^†^ 1.60 ± 1.04 ^†^	2.17 ± 1.64 ^†^ 2.56 ± 1.4 ^†^ 1.69 ± 1.12 1.50 ± 4.24 ^†^	2.22 ± 1.71 * 2.66 ± 1.31 * 1.68 ± 1.10 * 1.55 ± 3.04 *	U/16	U/16
**Gangwar et al., 2018**	6	14	1.36 ± 0.42	1.45 ± 0.45	1.41 ± 0.43 *	13	1.90 ± 0.43	2.14 ± 0.52	2.02 ± 0.48 *	U/14	U/13
										(90%)	
**Khan et al., 2018**	4–5 6 7–8 10–11 12 13–14	17	0.34 ± 0.49 ^†^ 0.44 ± 0.52 * 0.53 ± 0.54 ^†^ 0.66 ± 0.62 ^†^ 0.72 ± 0.65 * 0.78 ± 0.7 ^†^	0.37 ±0.49 ^†^ 0.48 ± 0.54 * 0.59 ±0.58 ^†^ 0.70 ±0.62 ^†^ 0.76 ± 0.65 * 0.82 ± 0.7 ^†^	0.36 ± 0.48 * 0.46 ± 0.52 * 0.56 ± 0.55 * 0.68 ± 0.61 * 0.74 ± 0.64 * 0.8 ± 0.69 *	16	0.74 ± 0.48 ^†^ 0.82 ± 0.48 * 0.89 ± 0.48 ^†^ 1.00 ± 0.48 ^†^ 1.07 ± 0.46 * 1.14 ± 0.44 ^†^	0.83 ± 0.56 ^†^ 0.92 ± 0.54 * 1.00 ± 0.52 ^†^ 1.10 ± 0.48 ^†^ 1.18 ± 0.48 * 1.25 ± 0.48 ^†^	0.79 ± 0.52 * 0.87 ± 0.5 * 0.95 ± 0.5 * 1.05 ± 0.48 * 1.12 ± 0.46 * 1.2 ± 0.46 *	17/17 (100%)	16/16 (100%)
**Öncü et al., 2019**	12	30	-		0.7 ± 0.5	30	-		1.3 ± 0.6	30/30 (100%)	30/30 (100%)
**Soni et al., 2020**	4	8	−0.03 ± 0.56	−0.18 ± 0.3	−0.11 ± 0.44 *	8	−0.09 ± 0.76	−0.17 ± 0.42	−0.13 ± 0.6 *	8/8 (100%)	8/8 (100%)
**Alam et al., 2020**	3 6 12 ^+^	10	3.18 ± 2.36 2.03 ± 0.95 1.25 ± 0.85	2.19 ± 1.75 1.38 ± 0.64 0.99 ± 0.66	2.69 ± 2.09 * 1.71 ± 0.86 * 1.12 ± 0.74 *	10	2.57 ± 0.94 1.83 ± 0.46 0.86 ± 0.71	1.96 ± 1.27 1.56 ± 1.1 0.58 ± 0.79	2.27 ± 1.13 * 1.7 ± 0.83 * 0.72 ± 0.74 *	100%	100%
**Abdel-Rahman et al., 2020**	6 12 18	7	0.36 ± 0.32 0.49 ± 0.36 0.54 ± 0.32	0.47 ± 0.38 0.6 ± 0.44 0.67 ± 0.49	0.42 ± 0.34 * 0.55 ± 0.39 * 0.61 ± 0.4 *	7	0.16 ± 0.08 0.24 ± 0.09 0.23 ± 0.09	0.2 ± 0.06 0.21 ± 0.07 0.24 ± 0.08	0.18 ± 0.07 * 0.23 ± 0.08 * 0.24 ± 0.08 *	7/7 (100%)	7/7 (100%)

IIP: immediate implant procedure; U: unspecified; mm: millimeters; SD: standard deviation; *: estimated values obtained using the formulae for combining groups; ^†^: SD calculated with the standard error of the mean (SEM); ^+^: three patients were lost to follow-up at 12 months (n = 7).

**Table 5:** Risk-of-bias assessment of the controlled clinical trials included.

Author, Year	Possible Source of Bias (Type of Bias)							
	Random Sequence Generation (Selection)	Allocation Concealment (Selection)	Blinding of Participants and Personnel (Performance)	Blinding of Outcome Assessment (Detection)	Incomplete Outcome Data (Attrition)	Selective Reporting (Reporting)	Other Bias	Overall Assessment
**Al Nashar et al., 2015**	Unclear risk	High risk	High risk	Low risk	Low risk	Low risk	Low risk	High risk
**ArRejaie et al., 2016**	Low risk	Low risk	High risk	Unclear risk	Unclear risk	Unclear risk	Low risk	High risk
**Gangwar et al., 2018**	Low risk	Unclear risk	High risk	Unclear risk	Unclear risk	Unclear risk	Low risk	High risk
**Khan et al., 2018**	Low risk	Low risk	High risk	Low risk	Low risk	Low risk	Low risk	High risk
**Öncü et al., 2019**	Low risk	Unclear risk	High risk	Unclear risk	Low risk	Low risk	Low risk	High risk
**Soni et al., 2020**	Low risk	Unclear risk	High risk	Unclear risk	Low risk	High risk	Low risk	High risk
**Alam et al., 2020**	Low risk	Unclear risk	High risk	Low risk	Unclear risk	Low risk	Low risk	High risk
**Abdel-Rahman et al., 2020**	Low risk	Unclear risk	High risk	Unclear risk	Low risk	Low risk	Low risk	High risk

## Data Availability

Data sharing not applicable.

## Supplementary Materials

The following are available online at https://www.mdpi.com/article/10.3390/ma14164582/s1, Figure S1: Fixed-effect meta-analysis evaluating MBL in both aspects at 6 months in immediate implants procedures with PCs vs. no PCs. (MD −0.50, 95% CI [−0.57, −0.43]; *p* < 0.00001). (I^2^ = 0%), Figure S2: Fixed-effect meta-analysis evaluating MBL in both aspects at 6 months in immediate implants procedures with PCs vs. no PCs. (SMD −1.45, 95% CI [−1.96, −0.94]; *p* < 0.00001). (I^2^ = 91%), Figure S3: Random-effect meta-analysis evaluating MBL in both aspects at 6 months in immediate implants procedures with PCs vs. no PCs. (SMD −2.19, 95% CI [−4.07, −0.31]; *p* = 0.02). (I^2^ = 91%), Figure S4: Fixed-effect meta-analysis evaluating MBL in both aspects at 12 months in immediate implants procedures with PCs vs. no PCs. (SMD −1.22, 95% CI [−1.63, −0.80]; *p* < 0.00001). (I^2^ = 92%), Figure S5: Fixed-effect meta-analysis evaluating MBL in both aspects at 12 months in immediate implants procedures with PCs vs. no PCs. (MD −0.5, 95% CI [−0.57, −0.43]; *p* < 0.00001). (I^2^ = 0%), Figure S6: Random-effect meta-analysis evaluating MBL in both aspects at 12 months in immediate implants procedures with PCs vs. no PCs. (SMD −2.02, 95% CI [−3.86, −0.35]; *p* = 0.02). (I^2^ = 92%), Figure S7: Fixed-effect meta-analysis evaluating MBL in both aspects at 12 months in immediate implants procedures with PCs + bone graft group vs. only bone graft. (MD 0.10, 95% CI [−0.14, 0.33]; *p* = 0.42). (I^2^ = 84%), Figure S8: Fixed-effect meta-analysis evaluating MBL in both aspects at 12 months in immediate implants procedures with PCs + bone graft group vs. only bone graft. (SMD −0.30, 95% CI [−0.80, 0.21]; *p* = 0.25). (I^2^ = 78%), Figure S9: Random-effect meta-analysis evaluating MBL in both aspects at 12 months in immediate implants procedures with PCs + bone graft group vs. only bone graft. (SMD −0.1, 95% CI [−1.21, 1.00]; *p* = 0.85). (I^2^ = 78%), Figure S10: Fixed-effect meta-analysis evaluating MBL in both aspects at 12 months in immediate implants procedures with PCs + bone graft group vs. only bone graft. (MD 0.31, 95% CI [0.03, 0.58]; *p* = 0.03). (I^2^ = 0%), Figure S11: Random-effect meta-analysis evaluating MBL in both aspects at 12 months in immediate implants procedures with PCs + bone graft group vs. only bone graft. (SMD 0.33, 95% CI [−0.48, 1.14]; *p* = 0.43). (I^2^ = 53%), Figure S12: Fixed-effect meta-analysis evaluating MBL in both aspects at 12 months in immediate implants procedures with PCs + bone graft group vs. only bone graft. (SMD 0.18, 95% CI [−0.34, 0.70]; *p* = 0.49). (I^2^ = 53%).

## Abbreviations

IIP	immediate implant procedures
PCs	platelet concentrates
MBL	marginal bone loss
PRF	platelet-rich fibrin
PRP	platelet-rich plasma
L-PRF	leucocyte platelet-rich fibrin
L-PRP	leucocyte platelet-rich plasma
P-PRF	pure platelet-rich fibrin
P-PRP	pure platelet-rich plasma
PRGF	plasma rich in growth factors
PICO	population, intervention, comparison, outcome
RCT	randomized controlled trial
CCT	clinical controlled trial
SD	standard deviation
SE	standard error
