# Supplementary material for: Effect of Platelet Concentrates on Marginal Bone Loss of Immediate Implant Procedures: A Systematic Review and Meta-Analysis
Source: Materials (Basel). 2021 Aug 15;14(16):4582. doi: 10.3390/ma14164582 (PMC8401659; doi:10.3390/ma14164582)
Supplement: Supplementary file 1 [file materials-14-04582-s001.zip › materials-1225210-supplementary.pdf]

Supplementary material

MBL at 6 months in PCs vs. no PCs group

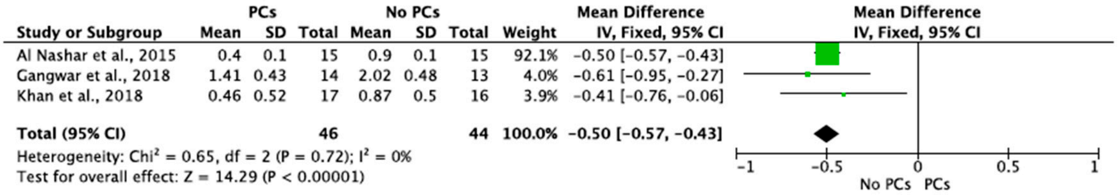

Figure S1. Fixed-effect meta-analysis evaluating MBL in both aspects at 6 months in immediate implants procedures with PCs vs. no PCs. (MD −0.50, 95% CI [−0.57, −0.43]; $p < 0.00001$). ($I^2 = 0\%$).

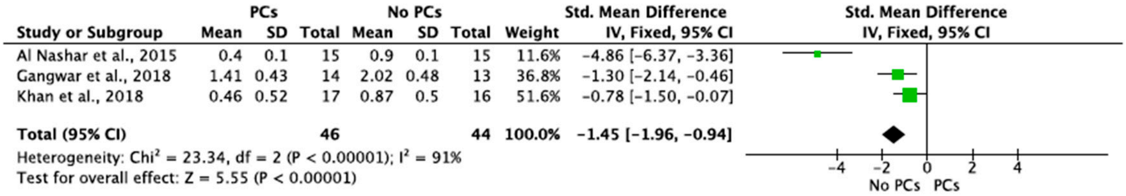

Figure S2. Fixed-effect meta-analysis evaluating MBL in both aspects at 6 months in immediate implants procedures with PCs vs. no PCs. (SMD −1.45, 95% CI [−1.96, −0.94]; $p < 0.00001$). ($I^2 = 91\%$).

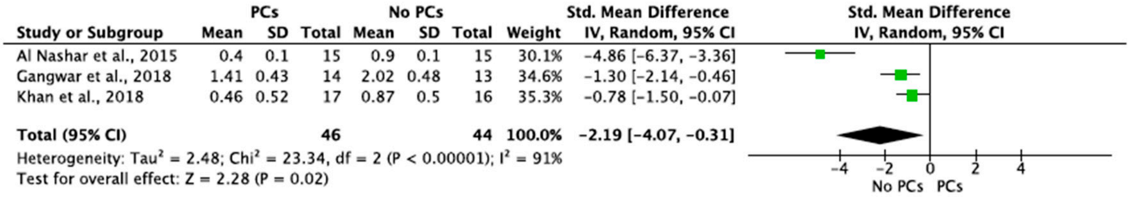

Figure S3. Random-effect meta-analysis evaluating MBL in both aspects at 6 months in immediate implants procedures with PCs vs. no PCs. (SMD −2.19, 95% CI [−4.07, −0.31]; $p = 0.02$). ($I^2 = 91\%$).

MBL at 12 months in PCs vs. no PCs:

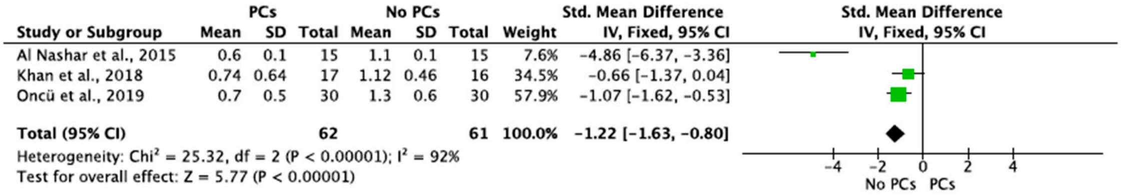

Figure S4. Fixed-effect meta-analysis evaluating MBL in both aspects at 12 months in immediate implants procedures with PCs vs. no PCs. (SMD −1.22, 95% CI [−1.63, −0.80]; $p < 0.00001$). ($I^2 = 92\%$).

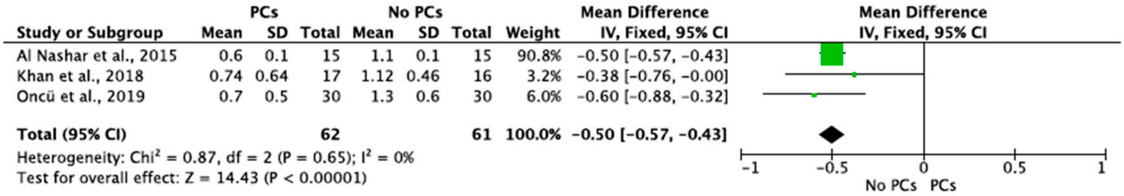

Figure S5. Fixed-effect meta-analysis evaluating MBL in both aspects at 12 months in immediate implants procedures with PCs vs. no PCs. (MD −0.5, 95% CI [−0.57, −0.43]; $p < 0.00001$). ($I^2 = 0\%$).

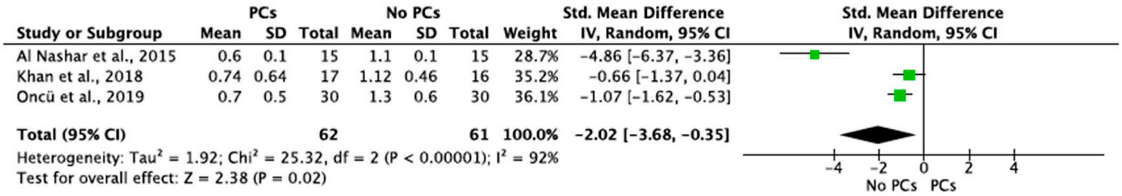

Figure S6. Random-effect meta-analysis evaluating MBL in both aspects at 12 months in immediate implants procedures with PCs vs. no PCs. (SMD −2.02, 95% CI [−3.86, −0.35]; $p = 0.02$). ($I^2 = 92\%$).

MBL at 6 months in PCs + bone graft vs. only bone graft:

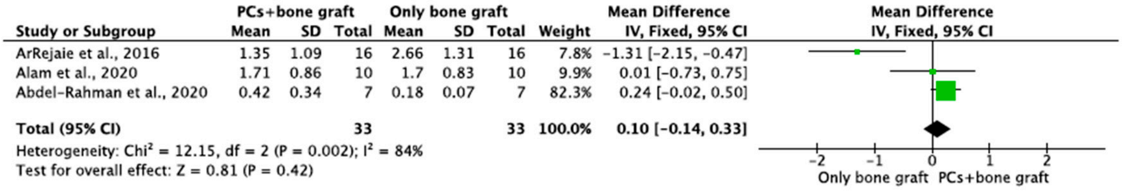

Figure S7. Fixed-effect meta-analysis evaluating MBL in both aspects at 12 months in immediate implants procedures with PCs + bone graft group vs. only bone graft. (MD 0.10, 95% CI [−0.14, 0.33]; $p = 0.42$). ($I^2 = 84\%$).

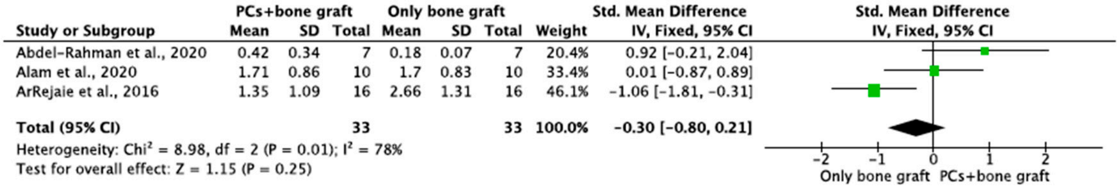

Figure S8. Fixed-effect meta-analysis evaluating MBL in both aspects at 12 months in immediate implants procedures with PCs + bone graft group vs. only bone graft. (SMD −0.30, 95% CI [−0.80, 0.21]; $p = 0.25$). ($I^2 = 78\%$).

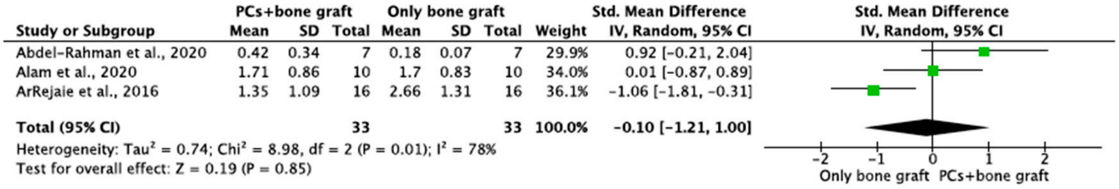

Figure S9. Random-effect meta-analysis evaluating MBL in both aspects at 12 months in immediate implants procedures with PCs + bone graft group vs. only bone graft. (SMD −0.1, 95% CI [−1.21, 1.00]; $p = 0.85$). ($I^2 = 78\%$).

MBL at 12 months in PCs + bone graft vs. only bone graft:

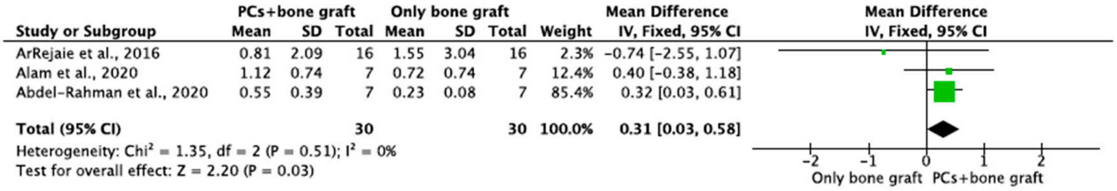

Figure S10. Fixed-effect meta-analysis evaluating MBL in both aspects at 12 months in immediate implants procedures with PCs + bone graft group vs. only bone graft. (MD 0.31, 95% CI [0.03, 0.58]; $p = 0.03$). ($I^2 = 0\%$).

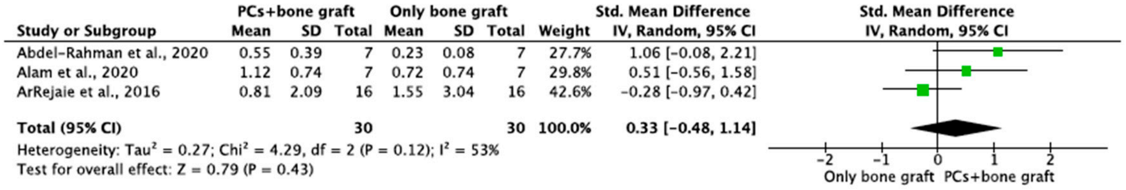

Study or Subgroup	PCs+bone graft Mean	SD	Total	Only bone graft Mean	SD	Total	Weight	Std. Mean Difference IV, Random, 95% CI
Abdel-Rahman et al., 2020	0.55	0.39	7	0.23	0.08	7	27.7%	1.06 [−0.08, 2.21]
Alam et al., 2020	1.12	0.74	7	0.72	0.74	7	29.8%	0.51 [−0.56, 1.58]
ArRejaie et al., 2016	0.81	2.09	16	1.55	3.04	16	42.6%	−0.28 [−0.97, 0.42]
**Total (95% CI)**			**30**			**30**	**100.0%**	**0.33 [−0.48, 1.14]**

Heterogeneity: Tau² = 0.27; Chi² = 4.29, df = 2 (P = 0.12); I² = 53%
Test for overall effect: Z = 0.79 (P = 0.43)

Figure S11. Random-effect meta-analysis evaluating MBL in both aspects at 12 months in immediate implants procedures with PCs + bone graft group vs. only bone graft. (SMD 0.33, 95% CI [−0.48, 1.14]; $p = 0.43$). ($I^2 = 53\%$).

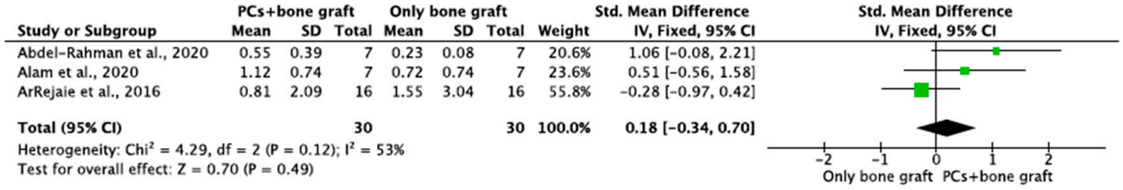

Study or Subgroup	PCs+bone graft Mean	SD	Total	Only bone graft Mean	SD	Total	Weight	Std. Mean Difference IV, Fixed, 95% CI
Abdel-Rahman et al., 2020	0.55	0.39	7	0.23	0.08	7	20.6%	1.06 [−0.08, 2.21]
Alam et al., 2020	1.12	0.74	7	0.72	0.74	7	23.6%	0.51 [−0.56, 1.58]
ArRejaie et al., 2016	0.81	2.09	16	1.55	3.04	16	55.8%	−0.28 [−0.97, 0.42]
**Total (95% CI)**			**30**			**30**	**100.0%**	**0.18 [−0.34, 0.70]**

Heterogeneity: Chi² = 4.29, df = 2 (P = 0.12); I² = 53%
Test for overall effect: Z = 0.70 (P = 0.49)

Figure S12. Fixed-effect meta-analysis evaluating MBL in both aspects at 12 months in immediate implants procedures with PCs + bone graft group vs. only bone graft. (SMD 0.18, 95% CI [−0.34, 0.70]; $p = 0.49$). ($I^2 = 53\%$).
